# Attitudes toward the integration of nutritional assessment and counseling in the framework of physical therapy: a cross-sectional survey

**DOI:** 10.1186/s12909-023-04706-2

**Published:** 2023-10-26

**Authors:** Roy Netzer, Michal Elboim-Gabyzon

**Affiliations:** Department of Physical Therapy, School of Social Welfare and Health Sciences, University of Haifa, 199 Aba Khoushy Ave., Mount Carmel, Haifa, Israel

**Keywords:** Attitudes, Registered physical therapists, Nutrition, Assessment, Integration, Nutritional knowledge

## Abstract

**Background:**

As the field of physical therapy increasingly acknowledges the integral role of nutritional assessment and counseling, revisiting this subject is essential due to limited updated information regarding this topic. Furthermore, it is crucial to investigate the factors that may influence physical therapists’ attitudes toward integrating nutritional assessment and counseling into their physical therapy practice. The study’s primary aim was to explore the attitudes of registered physical therapists in Israel toward incorporating nutritional assessment and counseling into their clinical practice. We also explored the relation between personal and professional characteristics of physical therapists and their attitudes.

**Methods:**

An anonymous nationwide online self-report survey was conducted. It included three sections: personal and professional background characteristics, self-reported level of nutritional knowledge and sources of nutritional knowledge, and attitudes toward incorporating nutritional assessment and counseling into practice. Descriptive statistics were calculated for all outcome measures. The total score of the attitude statements was compared between different groups of participants. Multiple linear regression analysis was used to predict positive attitudes.

**Results:**

The study included 409 physical therapists. The attitude score was 19.78 (2.53) of 25, indicating a positive attitude. A majority (67%) reported that they did not acquire knowledge regarding nutrition during their entry-level studies or in other formal settings (63%). The three primary sources of nutritional information for the participants reported were the Internet (87.0%), registered dietitian (70.0%), and professional, scientific journals (36.0%). Prior knowledge acquired during entry-level physical therapist studies and clinical experience of ≥ 13 years significantly predicted positive attitudes (β = 0.17, β = −0.13, respectively).

**Conclusion:**

Registered physical therapists held a positive attitude toward incorporating nutritional assessment and counseling into their clinical practice. Two background professional characteristics were predictors of positive attitudes. Accordingly, there is need to appropriate formal education regarding nutritional issues. Further studies are required to explore the actual integration of nutrition into the physical therapy framework.

## Introduction

An unhealthy lifestyle, which is characterized by poor eating habits, sedentary lifestyle, physical inactivity, smoking, and lack of sleep, is associated with ischemic heart disease, smoking-related conditions, hypertension and stroke, obesity, type 2 diabetes, and cancer [1–3]. Accordingly; these health conditions sometimes indicate lifestyle diseases that have a high impact on healthcare services and the individual [4, 5].

The World Health Organization (WHO) and the Centers for Disease Control and Prevention recently emphasized that all healthcare professionals must promote healthy patient lifestyles [6]. Accordingly, physical therapy is expected to play a role in health promotion. Although addressing the individual’s level of physical activity is inherent to physical therapy, it is argued that other aspects of the individual’s lifestyle should be addressed during the physical therapy clinical encounters. One aspect of a healthy lifestyle that has elicited increased interest in the physical therapy literature is the promotion of healthy eating habits [5–8].

A recent position statement by the American Physical Therapy Association (APTA) stated that nutrition issues are within the scope of professional practice because “Diet and Nutrition are key components of primary, secondary, and tertiary prevention of many conditions managed by physical therapists” [6]. Accordingly, the physical therapist should include nutrition issues as part of their assessment and provide appropriate consultation. Thus, for example, in treating osteoporosis and related pathological fractures, the physical therapist typically prescribes a physical activity program to maintain bone health. However, it is vital that the physical therapist also evaluate the patient’s nutritional status, particularly the daily intake of calcium and vitamin D [9]. Moreover, the physical therapist must be aware when referral to a registered dietitian is necessary [10]. Thus recognition of basic dietary counseling as an integral component of clinical readiness for physical therapists is on the rise, with an emphasis on assessing and managing the prevention of non-communicable diseases, and lifestyle-related chronic conditions [7, 11, 12]. Clinical preparedness of physical therapists involves a triad consisting of positive attitude toward the subject, adequate knowledge, and the relevant skills [13]. Consequently, there is a need to explore the attitudes of registered physical therapists toward integrating nutrition issues. Most studies exploring the attitudes of registered physical therapists toward integrating nutrition issues as part of their professional role were published over 20 years ago [8, 14–17]. Considering the recent emphasis that nutritional assessment and counseling are within the scope of the physical therapist’s profession [6], there is a need to revisit this issue. Accordingly, the current study examines the attitudes of physical therapists toward nutritional aspects in Israel, a representative country of a developed country with advanced physical therapy services in the Middle East [18]. Additionally, the current study is relevant because there are countries that are similar to Israel in terms of multinationalism, various religious communities, and a high percentage of immigrants [19].

Another limitation of the existing literature is the lack of distinction between the work settings of physical therapists. For example, two previous studies demonstrated that physical therapists working with sports injuries had significantly different attitudes toward the integration of nutritional issues during treatment compared to physical therapists working in acute care [14, 16]. Thus, it is vital to investigate the factors that may affect the attitudes of physical therapists toward integrating nutritional assessment and counseling as part of the physiotherapy they provide [8]. Furthermore, it is essential to concurrently investigate not only the attitudes but also the level of knowledge concerning nutrition issues and its sources. An examination of the relationship between these factors is imperative since it has been identified as a component of clinical readiness [13] and a determinant influencing the actual integration of nutritional assessment and counseling into physical therapy treatment. This remains true even in the presence of positive attitudes toward this matter [7, 11].

Therefore, the current study aimed to explore (1) the attitudes of registered physical therapists toward incorporating nutritional assessment and counseling into their clinical practice, (2) the level and sources of knowledge regarding nutritional issues, and (3) what background personal and professional characteristics of physical therapists are related to their attitudes toward the integration of nutrition in the framework of physical therapy.

## Methods

### Study design and setting

An anonymous nationwide online self-report survey using “Qualtrics” software was conducted between March 20, 2022, and July 17, 2022.

This study was approved by the Ethics Committee at the Faculty of Social and Health Sciences, University of Haifa (case number 2858). Accompanying online instructions indicated that completing and submitting the questionnaire indicated informed consent.

### Participants

Participant recruitment involved the use of social media channels such as Facebook, physical therapy professional groups, the newsletter of the Israeli Physiotherapy Society, and word of mouth. The study included registered physical therapists with at least 1 year of experience, excluding those with an academic degree in nutrition.

### Study procedure

Phase A–The first stage involved the development of an assessment tool in Hebrew based on the questionnaire “Knowledge and Attitudes of Nutrition, Nursing, Physical Therapy, and Exercise Physiology / Physical Activity & Fitness Majors, regarding Nutrition,” which was developed by Thomas et al. [17]. The original questionnaire was modified to adapt it to the physical therapist population in Israel, the update evidence-based nutritional guidelines, and the Israel Ministry of Health 2020’s position paper regarding optimal nutrition in various populations [9, 20]. The questionnaire was then sent to five registered physical therapists, three registered dietitians, and three physical therapy students for an initial assessment. Based on their comments, (1) the option “do not know” was added to multiple choice questions to lower the chance that responders will guess, and (2) the number of questions was reduced from 83 to 60.

Phase B–The final version was transformed to the “Qualtrics” software format.

Phase C –The questionnaire was distributed among the physical therapists in the State of Israel.

### Outcome measures

The current paper presents the results of only the first part (out of two) of the questionnaire, which includes the following three sections:

Section A: Questions regarding personal and professional details, including age, height, weight, academic degrees, years of clinical experience, place of work, and previous self-nutrition counseling.

Section B: Self-report on the level and sources of knowledge regarding nutritional issues as determined by questions referring to previous exposure to this topic during undergraduate studies or in other educational settings; the three primary sources used to obtain nutritional information; and the three primary sources ignored when seeking nutrition information. Additionally, the physical therapists’ level of confidence regarding their level of knowledge was determined by two statements: (1) “I have sufficient knowledge to perform an initial nutritional review as part of the patient’s initial medical history” and (2) “During my undergraduate degree, I learned when to refer a patient for dietary advice.” The responders had to rate their agreement with these statements on a five-point Likert-type scale ranging from 1 (strongly disagree) to 5 (strongly agree).

Section C: Determining the participants’ attitudes toward incorporating nutritional assessment and counseling into their practice using five statements rated on a five-point Likert-type scale from 1 (strongly disagree) to 5 (strongly agree).

### Statistical analysis

Descriptive statistics were calculated for all the outcome measures obtained in Sections A and B. Additionally, a total score, ranging from 5 to 25, regarding the participants’ attitudes toward incorporating nutritional assessment and counseling into their practice was calculated from the five statements in Section C. Higher scores indicated a more favorable attitude toward incorporating nutritional assessment and counseling into practice. Internal consistency of the attitude statements was calculated using Cronbach’s alpha, with α values ≥ 0.9 considered excellent, 0.7 ≤ α < 0.9 good, 0.6 ≤ α < 0.7 acceptable, 0.5 ≤ α < 0.6 poor, and values of α < 0.5 unacceptable [22].

The total score of the attitude statements was compared between different groups of participants as follows: (1) three groups differing in their professional seniority as determined by years of practice (1–2, 3–12, > 13 years); (2) four groups practicing in different work settings (outpatient clinic, acute care hospital, rehabilitation hospital/nursing home, and child development clinics); (3) prior self-referral for nutritional counseling (yes or no); (4) knowledge acquired regarding nutrition during their first degree (yes or no); and (5) knowledge acquired regarding nutrition in other settings (yes or no). A comparative analysis was performed using the T-test for independent samples when two groups were involved and one-way analysis of variance to compare more than two groups.

Bivariate correlation coefficient analysis was utilized to determine the relationship between the attitude scores of physical therapists and the following variables: age and body mass index (BMI) (Pearson correlation) and the level of confidence concerning the nutritional knowledge of physical therapists (Spearman correlation). Correlation coefficients of 0.10–0.29, 0.30–0.49, and 0.50 indicated small, moderate, and large effect sizes, respectively [21].

Multiple linear regression analysis was used to predict positive attitudes toward integrating nutritional assessment and counseling in the framework of physical therapy by years of clinical experience, workplace setting, prior knowledge regarding nutrition obtained in undergraduate studies, and BMI. Significance was established at p-values ≤ 0.05. Analysis was preformed using SAS version 9.4 software.

## Results

The total number of respondents was 426. We excluded four who did not meet the inclusion criteria and 13 who reported that their principal workplace was the academia and who seemed unengaged in clinical practice. Thus, 409 participants were included in the study.

### Personal and professional background characteristics

The background characteristics of the participants are summarized in Table 1. The mean (standard deviation) age and BMI of the participants were 41.2 (10.0) years and 24.1 (3.6) kg/m^2^, respectively. Most participants studied in Israel (94.3%), and most (92.6%) had ≥ 3 years of professional experience. The majority of the participants (63.3%) practiced in outpatient clinics. Almost half of the responders reported that they referred themselves at least once for nutritional counseling.

**Table 1:** Background characteristics

Characteristics		Participants (N = 409)
Age, years, mean (SD)		41.2 (10.0)
BMI, kg/meter^2^, mean (SD)		24.1 (3.6)
Additional degree/student for an additional degree other than a bachelor’s degree in physical therapy? Yes, n (%)	Bachelor’s degree (not in physical therapy)	23 (5.6)
	Master’s degree (in physical therapy/not in physical therapy)	121 (29.5)
	Doctorate (in physical therapy/ not in physical therapy)	5 (1.2)
	None	260 (63.5)
Professional seniority n (%)	1–2 years	30 (7.3)
	3–12 years	191 (46.7)
	≥ 13 years	188 (45.9)
Location of the university of the entire PT program? n (%)	Israel	386 (94.3)
	Abroad	23 (5.6)
Main workplace set n (%) *	Outpatient clinic	258 (63.3)
	Acute hospitals	48 (11.7)
	Rehabilitation hospital/nursing home	48(11.7)
	Child development	53 (13.0)
Previous self-referral/applied for nutritional counseling, n (%)	Yes	198 (48.4)
	No	211 (51.5)

* Two missing

SD, standard deviation; BMI, body mass index; n, number; %, percentage; PT, Physical Therapy

### Knowledge level, self-confidence, and sources of knowledge

67% reported that they did not acquire knowledge regarding nutrition during their first-degree studies of physical therapy or in other settings (63.0%).

The physical therapists’ level of confidence regarding their nutritional knowledge was low; 60.0% of the participants reported that they did not have sufficient knowledge to perform an initial nutritional review, and only 6.0% reported that they learned when to refer a patient for dietary advice during their undergraduate degree. The Internet (87.3%), consultation with a registered dietician (70.2%), and a professional, scientific journal (36.0%) were the three primary sources of information participants relied on for nutritional information. The three sources of information least relied on were TV/Radio (57.5%), their own parents (48.0%), and newspaper/magazine (41.8%) (Table 2).

**Table 2:** Self-reported level and sources of knowledge and self-reported level of confidence regarding knowledge

Item		Participants (N = 409)
Prior to acquiring knowledge in nutrition during the entire degree? n (%)	Yes	73 (17.8)
	No	274 (66. 9)
	Don’t remember	62 (15.1)
Prior to acquiring knowledge of nutrition in other settings? n (%)	Yes	152 (37.1)
	No	257 (62.8)
I have sufficient knowledge to perform an initial nutritional review to assess nutritional status as part of the patient’s initial assessment. n (%)	Strongly disagree	103 (25.1)
	Disagree	143 (34.9)
	Unsure	118 (28.8)
	Agree	37 (9.0)
	Strongly agree	8 (1.9)
During my undergraduate degree, I learned when to refer a patient for dietary advice. n (%)	Strongly disagree	130 (31.7)
	Disagree	128 (31.3)
	Not sure	126 (30.8)
	Agree	19 (4. 6)
	Strongly agree	6 (1.4)
What are the three main sources that the participants rely on for receiving nutritional information? (%)**	Internet	87. 3
	Registered nutritionist	70. 2
	A professional scientific journal	35.9
	Newspaper/magazine	19.3
	Friends	19. 1
	A scientific book	15.7
	Other	14.9
	Doctors	12.7
	TV/Radio	8.8
	Personal trainer	5.6
	Introductory course to nutrition in the first degree	3. 7
	Parents	3.2
	Physiotherapist	2.7
	Nurse	0.98
What are the three sources that the participants least rely on for receiving nutritional information? (%)**		
	TV/radio	57.5
	Parents	47.9
	Newspaper/Magazine	41.8
	Friends	33.0
	Personal trainer	24.9
	Nurse	22.7
	Introductory course to nutrition in the first degree	15.9
	Doctors	13.5
	Physiotherapis	10.0
	Scientific book	9.8
	Professional scientific journal	7.8
	Internet	6.4
	Other	5.4
	Registered nutritionist	3.4

** The proportion of subjects who answered a certain category

n, number; %, percentage; TV, television

### Attitudes toward nutrition

Table 3 presents the total score of the attitudes scale and each of the five items. The mean and standard deviation of total score was 19.8 (2.5) (out of 25), indicating a high positive attitude toward incorporating nutritional assessment and counseling into practice. An acceptable internal consistency of the attitudes scale was demonstrated, with a Cronbach alpha reliability coefficient of 0.67.

**Table 3:** Attitudes toward the integration of nutritional assessment and counseling in the framework of physical therapy

Item	Mean(SD)
1. An introductory course on nutrition should be mandatory in the curriculum for bachelor’s degree in physical therapy	4.2 (0.8)
2. An introductory nutrition course should be given by a registered clinical nutritionist	4.5 (0.6)
3. Physiotherapists should have a good understanding of nutritional principles/recommendations	4.3 (0.6)
4. Rehabilitation process within the framework of physiotherapy must also include personal education for a balanced diet by the physiotherapist	2.7 (1.0)
5. Do you think physiotherapy should include reference to the nutritional status of the patient (by referring to a professional or by the physiotherapist)?	3.9 (0.7)
**Total attitudes toward the nutrition scale score**	**19**.**8 (2**.**5)**

SD, standard deviation

### Comparison of the attitudes score groups with different characteristics

Group comparisons are presented in Table 4. A significant difference was found in the mean total attitude score between groups with different clinical experiences. Participants with ≥ 13 years of experience demonstrated significantly more positive attitudes toward evaluating and counseling patients on nutritional issues. Participants who acquired knowledge regarding nutrition during their undergraduate physical therapist studies had more positive attitudes than those with no previous knowledge. The participants’ workplace and prior self-experience of nutritional counseling had no significant effect on their attitudes.

**Table 4:** Comparative analysis of the total attitudes toward nutrition score compared with background professional characteristics and prior knowledge

Variable		Total attitudes toward nutrition scale score, mean (SD)	Test statistic	p-value
Professional seniority	1–2 years	19.1 (2.5)	F(2,406) = 3.3	< 0.05
	3–12 years	19.5 (2.4)		
	≥ 13 years	20.1 (2.5)		
Main workplace	Private/health care institute	19. 7 (2.6)	F(3,403) = 0. 6	0. 6
	Acute care hospitals	19.6 (2.4)		
	Rehabilitation hospital/nursing home	19.7 (2.4)		
	Child development	20.2 (2.3)		
Prior referral/applied for nutritional counseling in the past	Yes	19.7 (2.6)	T (407) = − 0.3	0.7
	No	19.8 (2.4)		
Prior acquirement of knowledge in nutrition during first degree	Yes	20.4 (2.2)	T (345) = 2.6	< 0.01
	No	19.6 (2.5)		
Prior acquirement of knowledge in nutrition in other settings	Yes	20.0 (2.4)	T (407) = 1.7	0.08
	No	19.6 (2.5)		

### Bivariate correlation coefficient analysis

A significant low correlation was found between the mean total attitude score and the physical therapists’ confidence in self-knowledge necessary for assessing the patients’ nutritional status. No significant correlation was found between the mean total attitude score and age, BMI, and the physical therapists’ confidence regarding when to refer a patient for further dietary advice (Table 5).

**Table 5:** Bivariate correlation coefficient analysis between total attitudes toward nutrition scale, age, and BMI and the two statements regarding the level of confidence of the PT on their level of knowledge

Variable	Total attitudes toward nutrition scale score	
	Mean (SD)	r (p-value)
Age (years) ^a^	41.2 (10.0)	0.03 (0.5)
BMI ^a^	24.1 (3.5)	−0.002 (0.95)
I have sufficient knowledge to perform an initial nutritional review to assess nutritional status as part of the patient’s initial assessment. ^b^	2.2 (1)	0.23 (< 0.001)
During my undergraduate degree, I learned when to refer a patient for dietary advice. ^b^	2. 1 (0.9)	0.03 (0.5)

^a^Pearson correlation coefficient

^b^Spearman correlation coefficients

### Predictors of positive attitudes: a regression analysis

Table 6 presents the results of the multiple regression analysis. Prior knowledge acquired during entry-level physical therapist studies (β = 0.17, P < 0.01) and ≥ 13 years of clinical experience (β = −0.13, P < 0.05) significantly predicted positive attitudes toward integrating nutritional assessment and counseling in physical therapy. These two predictors explained 2.3% of the variance (R2 = 2.3, F (7.3) = 2.2, P < 0.05).

**Table 6:** Regression analysis of variable predictors for positive attitudes toward nutrition

Variable	Total attitudes toward nutrition scale score			
	Parameter estimate	Standard error	Standardized estimate	T value (p-value)
Professional seniority (1–2 years vs. >13 years)	−0.98	0.5	−0.1	−1.8 (0.06)
Professional seniority (3–12 years vs. >13 years)	−0.7	0.3	−0.1	−2.3 (0. 01)
Workplace – Acute hospital vs. private/health care institute	−0.1	0.4	−0.01	−0.3 (0.7)
Workplace – Rehabilitation hospital/nursing home vs. private/health care institute	0.002	0.4	0.0002	0.01 (0.99)
Workplace – Child development vs. private/health care institute	0.3	0.4	0.04	0. 8 (0.4)
Did you acquire knowledge in nutrition during your first degree? (Yes vs. no)	1.1	0.3	0.12	3.1 (0.001)
BMI	−0.0005	0. 03	0.0008	−0.02 (0.98)

F (7, 337) = 2.16, p = 0.03, R^2^ = 0.02

## Discussion

The current study demonstrated that registered physical therapists in Israel have a positive attitude toward incorporating nutrition assessment and counseling into their clinical practice (mean total score of 19.8/25). This result agrees with the APTA statement indicating that nutritional assessment and counseling are within the scope of the discipline [6]. It is also consistent with previous studies [5, 14–16], which demonstrated that practicing physical therapists agreed with the APTA statement as they had positive attitudes toward integrating nutritional assessment and counseling into their physical therapy.

In the current study, 67% of the participants reported that they had not received formal education regarding nutrition as part of their entry level education. These results are consistent with previous studies which have identified a deficiency in nutrition knowledge among physical therapists through objective testing [11, 14, 16, 17]. Nonetheless, to the best of our knowledge, this study stands out as the first to investigate the correlation between prior nutritional education during entry level studies and attitudes toward integrating nutrition into practice. The findings of the current study indicate that being exposed to nutrition courses during entry level studies predict positive attitudes toward integrating nutrition assessment and counseling into clinical practice. These findings compliment previous research that underscores the significance of attitudes and behavioral beliefs as factors influencing the actual integration of nutrition within the physical therapy treatment system [5]. This emphasizes the necessity to reevaluate the physical therapy curricula to ensure that appropriate content is incorporated. Moreover, it is imperative to establish continuing education programs that offer a range of seminars and workshop options for physical therapists who have completed their formal studies [5]. Furthermore, the content of nutrition-related studies should be standardized to ensure their contribution to enhancing patient outcomes and, consequently, improving the overall effectiveness of physical therapy. Improving knowledge related to nutritional issues is essential for positioning the profession at the forefront in the endeavor to mitigate lifestyle diseases and promote optimal health. [5].

Based on the current results, significant predictors of a positive attitude toward integrating nutritional assessment and counseling into physical therapy practice are a clinical experience of ≥ 13 years and prior knowledge acquired at the entry-level physical therapist education. To the best of our knowledge, no previous studies have examined the correlation between attitudes toward incorporating nutritional counseling and years of clinical practice, which was a positive predictor in the present study. In contrast, several studies have examined the correlation between attitudes toward incorporating nutritional counseling and prior knowledge. Similar to the present study, a previous study conducted among members of the Geriatric Section of the APTA showed a positive correlation between the two variables [15]. However, other studies conducted with physical therapists treating orthopedic and sports injuries [14] and those in the acute care setting [16] did not demonstrate a similar correlation. This discrepancy between the studies may be related to differences in the measurement tools used to determine the level of prior knowledge. Moreover, differences in the questions investigating the subjects’ attitudes (i.e., referring to the importance of acquiring nutritional knowledge, incorporating nutritional counseling, and/or referral to a dietician) may have also led to discrepancies.

Our hypothesis, based on previous studies [14, 16], was that the work setting of the physical therapists might contribute to the physical therapist’s attitude toward integrating nutrition into the treatment. However, this hypothesis was not substantiated in the present study, as no significant difference was found in the mean attitude score between different work settings. This result can be due to the unbalanced representation of different work settings, as a high percentage (63.3%) of the participants worked in outpatient clinics, while only 23.6% worked in acute hospitals/rehabilitation hospitals/ nursing homes.

This study presents a unique examination of the factors that predict favorable attitudes towards the incorporation of nutrition issues into physical therapy practice. We have identified two significant predictors for such positive attitudes: prior knowledge acquired during entry-level physical therapist studies (β = 0.17, P < 0.01) and having over 13 years of clinical experience (β = −0.13, P < 0.05). However, these predictors explained only about 2% of the variance for positive attitude. Notably, other factors such as age, body mass index and the level of confidence in nutritional knowledge which were tested did not correlate significantly with the participants’ attitudes. Given the absence of prior studies on this topic in the literature we are unable to compare our results with previous studies. Further research is needed to identify additional factors influencing these attitudes, such as aspects related to lifestyle and cultural background.The physical therapists demonstrated low confidence in their knowledge level which would enable them to assess the patients’ nutritional status and identify the need to refer them for dietary advice. This may be related to the fact that most responders did not take nutritional courses during their entry-level physical therapist education (67%) or in other educational settings (63.0%). This finding is supported by previous studies demonstrating that physical therapists’ lack of confidence can be improved by additional education on nutrition issues [5, 22, 23]. However, more studies should explore if the physical therapist’s low confidence can prevent the integration of nutrition into the physical therapy as noted in previous studies [5].

Examining the physical therapists’ source of knowledge on nutrition indicated that most did not receive formal education either during their entry-level physical therapist education (67.0%) or in other educational settings (63.0%). This finding should be noted by those who design the curricula for physical therapist study in Israel because physical therapists should provide accurate information regarding nutritional issues, correct patient misinformation, and refer patients to other health professionals when necessary. This is particularly vital when patients are directly referred to a physical therapist, as the encounter with the physical therapist may be the sole entry point into the health system [14].

The primary informal source for nutritional information noted by physical therapists in the current study was the Internet (87.3%), followed by consulting with registered dietitian (70.2%); only 36.0% referred to professional, scientific literature for information. These findings contradict previous findings [14–16], in which the primary source of information was the media and magazines. In contrast, in the current study, the media and newspapers/magazines were among the information sources least referred to for nutritional information. Our results may be related to the greater use of the Internet as a general source of information compared to 20 years ago when the cited references were conducted [14–16]. As the quality and accuracy of information obtained through the Internet is occasionally doubtful, nutrition studies must be integrated into entry-level and postgraduate physical therapy curricula, so that physical therapists can reliably include nutritional assessment and counseling within the physical therapy framework.

With the growing role of physical therapists in promoting wellness and a healthy lifestyle, determining their attitudes and knowledge regarding nutritional issues is vital. The current study, which included physical therapists from a wide range of specialties, is an important contribution to the literature.

### Limitation

Some study limitations must be noted. The survey was distributed via the Internet, so we could not calculate the response rate. Furthermore, the acquisition of nutrition knowledge during entry-level studies was self-assessed based on the participants’ memory. To mitigate this potential bias, we introduced the option to select “I do not remember”, as a possible answer to relevant questions. Future studies may consider incorporating objective knowledge assessments. However, even direct assessment of the respondents’ knowledge, their response as to source of their knowledge will rely on their recollections. Another limitation of this study is a selection bias, as individuals with a heightened interest in nutrition were probably more inclined to participate in the survey. All these limitations may potentially affect the generalizability of the current results.

## Conclusion

Registered physical therapists in Israel had a positive attitude toward incorporating nutritional assessment and counseling into their clinical practice. Acquisition of knowledge during entry-level physical therapist education and ≥ 13 years of clinical experience were predictors of a positive attitude. Most responders reported that they did not acquire knowledge regarding nutritional issues in their formal education. This indicates the need to update the physical therapist curriculum to allow them play an active role in patient education and health promotion. Further studies should explore whether physical therapists actually incorporate nutritional assessment and counseling in their clinical practice.

## Data Availability

The datasets used and/or analyzed during the current study available from the corresponding author on reasonable request.
